# Plasma Levels of Intact Parathyroid Hormone and Congestion Burden in Heart Failure: Clinical Correlations and Prognostic Role

**DOI:** 10.3390/jcdd9100334

**Published:** 2022-10-02

**Authors:** Pietro Scicchitano, Massimo Iacoviello, Andrea Passantino, Michele Gesualdo, Francesco Trotta, Marco Basile, Micaela De Palo, Piero Guida, Claudio Paolillo, Graziano Riccioni, Marco Matteo Ciccone, Pasquale Caldarola, Francesco Massari

**Affiliations:** 1 Cardiology Section, Hospital “F. Perinei”, 70022 Altamura, BA, Italy; 2 Cardiology Unit, Department of Medical and Surgical Sciences, University of Foggia, 71122 Foggia, FG, Italy; 3 Division of Cardiology and Cardiac Rehabilitation, Scientific Clinical Institutes Maugeri, IRCCS Institute of Bari, 70124 Bari, BA, Italy; 4 Cardiac Surgery Unit, Policlinic University Hospital, Piazza Giulio Cesare 11, 70124 Bari, BA, Italy; 5 Ospedale Generale Regionale “F. Miulli”, 70021 Acquaviva delle Fonti, BA, Italy; 6 Cardiology Section, Hospital “Umberto I”, 70033 Corato, BA, Italy; 7 Cardiology Unit, San Camillo de Lellis, Hospital, Via Isonzo 1, 71043 Manfredonia, FG, Italy; 8 Cardiology Unit, Policlinic University Hospital, Piazza Giulio Cesare 11, 70124 Bari, BA, Italy; 9 Cardiology Section, Hospital “S. Paolo”, 70123 Bari, BA, Italy

**Keywords:** heart failure, BIVA, BNP, PTH, prognosis, HYDRA score, congestion

## Abstract

Circulating parathyroid hormone (PTH) concentrations increase in heart failure (HF) and are related to disease severity. The relationship between PTH and congestion is still a matter of debate. The objective of this analysis was to evaluate the role of PTH as a marker of congestion and prognosis in HF. We enrolled 228 patients with HF. Intact PTH concentrations and HYDRA score (constituted by: B-type natriuretic peptide, blood urea nitrogen–creatinine ratio, estimated plasma volume status, and hydration status) were evaluated. The study endpoint was all-cause mortality. PTH levels were higher in acute compared with chronic HF and in patients with clinical signs of congestion (i.e., peripheral oedema and orthopnea). PTH concentrations significantly correlated with NYHA class and HYDRA score. At multivariate analysis of HYDRA score, estimated glomerular filtration rate (eGFR), and corrected serum calcium were independently determinants of PTH variability. Fifty patients (22%) died after a median follow-up of 408 days (interquartile range: 283–573). Using univariate Cox regression analysis, PTH concentrations were associated with mortality (hazard ratio [HR]: 1.003, optimal cut-off: >249 pg/mL—area under-the-curve = 0.64). Using multivariate Cox regression analysis, PTH was no longer associated with death, whereas HYDRA score, left ventricular ejection fraction, and eGFR acted as independent predictors for mortality (HR: 1.96, 0.97, and 0.98, respectively). Our study demonstrated that intact PTH was related to clinical and subclinical markers of congestion. However, intact PTH did not act as an independent determinant of all-cause death in HF patients.

## 1. Introduction

The parathyroid hormone (PTH) is one of the most important regulators of bone and mineral metabolism, mainly acting on calcium, phosphorus, and vitamin D reabsorption and excretion from bones, kidney, and gut [1]. The cardiovascular system is a target organ for PTH. Literature shows the influence of PTH on promotion of cardiac remodelling and fibrosis, cardiomyocytes hypertrophy, oxidative stress, increased activation of renin-angiotensin-aldosterone system (RAAS), arterial stiffness, and endothelial dysfunction [1,2,3,4].

The relationship between PTH and heart failure (HF) has been pointed out according to current evidence [5,6,7,8]. PTH has already been associated with cardiovascular and all-cause mortality in HF patients [9].

Intact PTH values > 65 pg/mL seemed to be associated with a 50% increased risk in incident HF in the Multi-Ethnic Study of Atherosclerosis (MESA) [10]. Data showed an increased rate of HF hospitalization in patients with higher PTH plasma levels [5,6], whereas decreased PTH concentrations might reduce HF exacerbation [11]. Specifically, a meta-analysis by Meng et al. demonstrated that higher PTH plasma concentrations were independently associated with HF decompensation [8]. On the other hand, Sugimoto et al. observed that baseline low-normal PTH values in patients with acute onset HF were related to all-cause death [12]. Therefore, PTH might be considered as a potential risk biomarker for HF onset and development, as well as for the overall survival of patients with HF [13,14].

Nevertheless, the in-depth evaluation of the possible association of PTH with congestion status in HF is still a matter of debate and there is scarce evidence in the current literature. Higher PTH plasma concentrations were related to worse New York Heart Association (NYHA) class [5,15,16,17,18,19], the increase in N-terminal Pro-B-Type Natriuretic Peptide (NT-pro BNP) [5,7,15,16,17,19,20,21] and BNP [19,21,22], the severity of lower extremity oedema [23], augmentation in pulmonary capillary wedge pressure, and reduction of stroke volume index/cardiac index [24]. No data actually exist about the possible link to other well-established congestion biomarkers in HF such as those derived from bioimpedance vector analysis (BIVA), intravascular signs of congestion such as plasma volume status, and/or venous markers of congestion such as blood urea nitrogen to creatinine ratio (BUN/Cr ratio) [25].

The aims of the present study were to evaluate the relationship between PTH and congestion biomarkers of HF and to consider the impact of PTH plasma concentrations on the overall prognosis of patients with both acute (AHF) and chronic (CHF) HF.

## 2. Materials and Methods

### 2.1. Study Populations

We enrolled 228 consecutive HF patients who were admitted to the Cardiology Unit of Hospital of Altamura–Bari (Italy) between January 2010 and November 2013 due to acute decompensation (AHF) or because they were followed-up as outpatients (CHF).

We collected clinical characteristics, blood chemistry data, BIVA, and pharmacological treatments from all patients. Left ventricular ejection fraction (LVEF) was calculated by means of echocardiography (Simpson’s method). BNP levels were assessed using a microparticle enzyme immunoassay (Architect, Abbott Park, IL, USA). The intra- and inter-assay variability coefficients ranged from 0.9% to 5.6% and 1.7% to 6.7%, respectively. Serum creatinine was measured with a Beckman Coulter AU 680 chemistry analyser. Intact serum PTH was determined by Electro-ChemiLuminescence Immunoassay Kit (Roche Diagnostics, Mannheim, Germany), with a normal range of 15–65 pg/mL and an inter-assay coefficient of variation ranging from 5.7 to 6.3%. All of these measurements were performed as a routine evaluation of the patients admitted to our department.

Inclusion criteria were: diagnosis of AHF or CHF, age > 18 years old, and willingness to be included in the study. Specifically, CHF was defined in case of the presence of specific signs (i.e., elevated jugular venous pressure, pulmonary crackles, and peripheral oedema) and symptoms (i.e., breathlessness, ankle swelling, and fatigue); echocardiographic features for definition of the type of CHF were adopted in relation to international guidelines [26]. Patients with AHF were defined in case of acute onset of signs and symptoms of HF which forced the need for urgent referral to the Emergency Department and access to intensive care units [26].

We considered as exclusion criteria all acute conditions, such as myocarditis, pericarditis, pulmonary embolism, acute coronary syndrome, and recent cardiac surgery intervention. We also excluded patients with cancer, end-stage kidney failure and/or haemodialysis, primary hyperparathyroidism, and systemic inflammatory diseases.

Patients were considered as suffering from coronary artery diseases if they experienced previous myocardial infarction and/or coronary revascularization (percutaneous and/or surgical revascularization); diabetes was defined as cases of fasting plasma glucose level ≥ 126 mg/dL or use of antidiabetics, whereas hypertension was identified by blood pressures higher than 140/90 mmHg.

The primary endpoint was all-cause mortality which was ascertained from available medical records or National Death Records.

The study complied with the Declaration of Helsinki and was approved by the local Institutional Review Board. Written informed consent was obtained from each patient at inclusion (protocol n. 0081801/CE–29 October 2015, study number: 4816).

### 2.2. Corrected Serum Calcium

To correct serum calcium concentration levels, we used the following equation: corrected calcium = total calcium (in mg/dL) + 0.8 × (4 − serum albumin [in g/dL]) [27].

### 2.3. Creatinine-Based Estimated Glomerular Filtration Rate (eGFR) Formula

We calculated estimated GFR using the Cockroft–Gault formula (mL/min/1.73 m^2^) as (140 − age in years) × (weight in Kg)/(72 × serum creatinine in mg/dL) × 0.85 (if female) [28]. According to the stages defined by the clinical guidelines of the National Kidney Foundation, the patients were categorized into four groups according to eGFR: <30 mL/min/1.73 m^2^, 30–59 mL/min/1.73 m^2^, 60–90 mL/min/1.73 m^2^, and >90 mL/min/1.73 m^2^.

### 2.4. BUN to Creatinine Ratio

BUN and serum creatinine were measured with a Beckman Coulter AU 680 chemistry analyser. The BUN/Cr median value in the general population is 15.0 (interquartile range [IQR]: 12.9–17.6) [29]. Evidence suggested that higher values of the BUN/Cr ratio are associated with “venous” congestion [30].

### 2.5. Estimated Plasma Volume Status

ePVS (dL/g) was calculated from haematocrit and haemoglobin values at admission using the Strauss–Duarte formula: (100 − haematocrit in %)/haemoglobin (in g/dL) [31].

### 2.6. Hydration Status Estimated by Bioimpedance Vector Analysis

BIVA was assessed on the right side of the body using tetrapolar impedance plethysmography that emitted a 50 kHz alternating sinusoidal current (CardioEFG, Akern RJL Systems, Florence, Italy) [32,33]. The instrument was calibrated each day by using a standard resistor supplied by the manufacturer (resistance [R] = 380 Ohm, reactance [Xc] = 47 Ohm, 1% error).

The two vector components, R and Xc, of BIVA were recorded and divided by the subject’s height. (R/Xc graph). The results were visualized as a BIVA-derived hydration percentage (hydration index, HI%). This value was calculated by an equation that used two components, R and Xc (Bodygram 1.4, Akern RJL Systems, Florence, Italy). The normal values are in the range between 72.7% and 74.3%, corresponding to the 50th percentile of the R/Xc graph [32].

### 2.7. Hydra Score

We previously demonstrated the prognostic ability of four markers related to the different components of congestion in HF: BNP (“haemodynamic” congestion), ePVS (“intravascular” congestion), hydration index BIVA-derived (“peripheral” hydration), and BUN/Cr (“venous” congestion). A simple score was generated based on the number of different elevated parameters of congestion: BNP > 441 pg/mL, BUN/Cr > 25, Duarte-PVS > 5.3dL/g and Hydration > 73.8% that we called the HYDRA score (range from 0 to 4) [25].

### 2.8. Statistical Analysis

Categorical variables were presented as percentage (%), and continuous variables were expressed as the mean and standard error of mean (SEM). Comparisons between two groups were performed with Student’s t-test or the Mann–Whitney U-test where appropriate. One-way analysis of variance (ANOVA) was used when comparisons were performed among more than two groups. The Spearman’s rho correlation coefficient was used to analyse the correlation between PTH levels and other variables. Multivariate regression analysis was utilized in order to assess the determinants of PTH levels. Receiver-operating characteristic (ROC) curve analysis was performed to calculate the area under the curve (AUC) values, and the optimal cut-off values for mortality were calculated as the point of maximum sensitivity and specificity.

Univariate and multivariate Cox proportional hazards regression models with estimations of hazard ratios (HR) and 95% confidence intervals (95% CI) were performed to evaluate the impact of variables on mortality. *p*-Values below 0.05 were defined as statistically significant.

Statistical analysis was performed using STATA software, version 12 (StataCorp, College Station, Tex, College Station, TX, USA).

## 3. Results

Two hundred and twenty-eight patients (mean age: 75 ± 0.7 years, 127 suffering with CHF and 101 with AHF) were included in this study. Table 1 summarizes the main characteristics of the study population.

**Figure 1:**
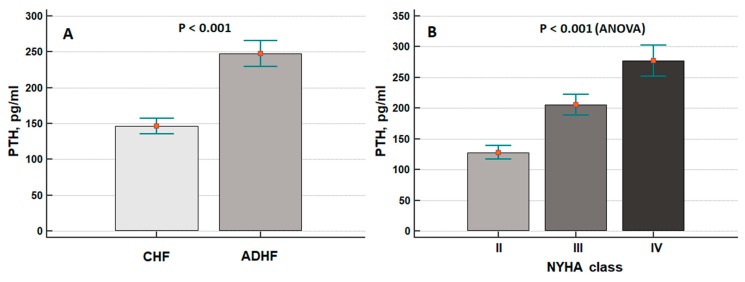
(**A**) Differences in parathyroid hormone (PTH) plasma concentrations between patients with acute (AHF) and chronic heart failure (CHF). (**B**) Relationship between PTH plasma concentrations and NYHA functional class (NYHA II: *n* = 100, NYHA III: *n* = 68, NYHA IV: *n* = 60). Data are expressed as mean ± standard error of mean.

Figure 1 demonstrates the variations in PTH plasma concentrations in patients with AHF and CHF (Figure 1A) and in relation to NYHA class (Figure 1B).

Patients with AHF showed significantly higher PTH plasma levels compared with uncongested patients.

**Figure 2:**
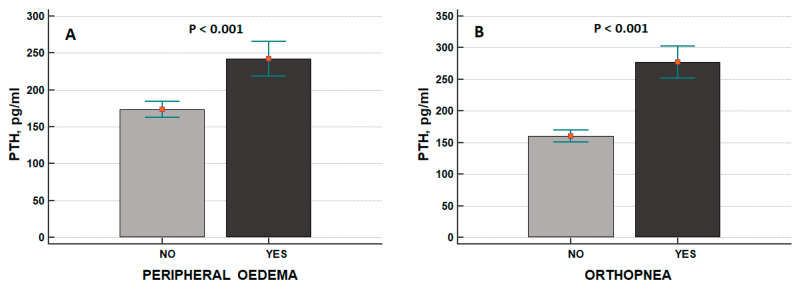
Relationship between intact parathyroid hormone (PTH) plasma concentrations and peripheral oedema (“no” peripheral oedema: *n* = 170, “yes” peripheral oedema: *n* = 58) (**A**) or orthopnoea (“no” orthopnoea: *n* = 171, “yes” orthopnoea: *n* = 57) (**B**). Data are expressed as mean ± standard error of mean.

These results were in line with the overt expression of clinical signs of congestion: patients with peripheral oedema or orthopnoea had higher levels of PTH compared with those who did not experience such signs (Figure 2A,B).

In addition, PTH levels significantly correlated to BNP levels (rho = 0.43), BUN/Cr (rho = 0.17), ePVS (rho = 0.23), and hydration status (rho = 0.43). At multivariate regression analysis, HYDRA score, corrected serum calcium, and eGFR remained significantly associated to PTH plasma levels (r = 0.33, r = −0.23 and r = −0.21, respectively, *p* < 0.01 for all). Nevertheless, PTH levels were correlated to HYDRA score (r = 0.28, *p*< 0.001), corrected serum calcium (r = −0.44, *p* < 0.001), and eGFR (r = −0.24, *p* < 0.001) in patients with AHF, whereas only HYDRA score remained significantly correlated to PTH plasma levels in patients with CHF (r = 0.27, *p* < 0.001).

**Figure 3:**
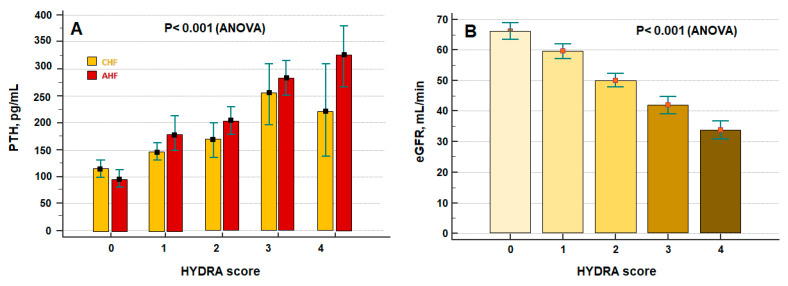
(**A**) Relationship between congestion degree as assessed by the HYDRA score (multiparametric approach: from 0 to 4) and parathyroid hormone (PTH) plasma concentrations in patients with chronic (CHF) and acute (AHF) heart failure. (**B**) Relationship between congestion degree as assessed by the HYDRA score (HYDRA “0”, *n* = 45, HYDRA “1”: *n* = 65, HYDRA “2”: *n* = 44, HYDRA “3”: *n* = 50, and HYDRA “4”: *n* = 24) and renal function (as assessed by means of estimated glomerular filtration rate [eGFR] calculated with Cockroft–Gault formula). Data are expressed as mean ± standard error of mean.

We evaluated the correlation between PTH and HYDRA score—which encompasses all of these variables in a unique score assessing the overall congestion status of patients with HF. Figure 3A depicts the relationship between PTH and progressive increase in HYDRA score in patients with AHF and CHF: the higher the HYDRA score, the higher the plasma concentrations of PTH (*p* < 0.001, ANOVA). Furthermore, the decrease in the eGFR values was related to the increase in HYDRA score (*p* < 0.001 ANOVA) (Figure 3B).

Similarly, PTH levels were inversely related to the corrected serum calcium plasma values (rho = −0.5, *p* < 0.001) and eGFR (rho = −0.35, *p* < 0.0001).

Fifty patients died at a median follow-up of 408 days (IQR: 283–573). The cumulative mortality incidence was 22%. Using univariate Cox regression analysis, PTH plasma levels, type of HF, age, NYHA class, LVEF, eGFR, and HYDRA score were predictors of mortality (Table 2).

**Figure 4:**
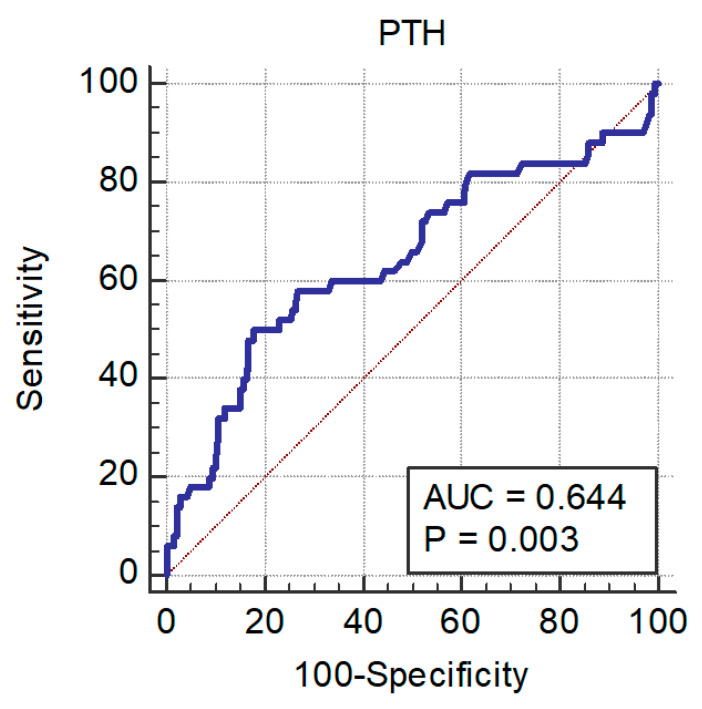
ROC curve for PTH as predictor of all-cause death. AUC: area-under-the curve; PTH: parathyroid hormone.

The analysis of ROC curves identified the cut-off of PTH as >249 pg/mL (AUC = 0.64, sensitivity 50% and specificity 82%, *p* = 0.003). Nevertheless, the multivariate Cox regression analysis revealed that PTH levels were no longer associated with mortality in HF patients, whereas HYDRA score, LVEF, and renal function still remained independent predictors for mortality (Table 2). Figure 4 represents the ROC curve for PTH.

## 4. Discussion

The role of PTH in cardiovascular diseases—and HF in particular—has been long debated, although definite data are lacking [1,2,4,5,6]. Our study tried to evaluate the prognostic role of intact PTH in patients with HF. The main results were the following: 1. Mean plasma concentrations of intact PHT are higher in AHF than CHF. 2. Intact PTH plasma concentrations are directly related to eGFR, and thus to kidney function. 3. PTH was not an independent predictor for all-cause mortality in patients with HF, whereas congestion status—as evaluated by the multiparametric HYDRA score—LVEF, and renal function still acted as independent predictors of mortality.

This is the first study that has tried to evaluate possible correlations between the congestion status of patients with HF and plasma concentrations of intact PTH. Notably, Zhang et al. [16] previously demonstrated that PTH was directly related to NYHA class and NT-proBNP. Similar results were derived from the analysis of Gruson et al. [19], although the 1–84 PTH assay—instead of intact PTH—was adopted in their analysis.

In addition, Altay et al. [22] confirmed the relationship between PTH and advanced HF as evaluated by means of NYHA class, but congestion markers such as BNP failed to be associated with PTH using multivariate regression analysis. The literature is scant about the possible interplay between PTH and congestion signs and symptoms. We observed higher PTH plasma concentrations in HF patients with peripheral oedema and orthopnoea (Figure 2A,B). When gathering together congestion markers into the HYDRA score—namely based on BNP concentrations, BUN/Cr ratio, Duarte-PVS, and Hydration Index as assessed by BIVA [25]—increased PTH values were found in patients with higher HYDRA scores (Figure 3A).

All of these associations remain a matter of debate as little data support the pathophysiological background about the correlation between PTH and congestion in HF. Hypotheses [34] considered the influence of renal impairment in HF as a determinant of the deregulation in PTH secretion and expression. For instance, venous congestion and systemic hypoperfusion might promote the occurrence of cardiorenal syndrome in patients with HF, and kidney dysfunction in patients with HF [35]. Kidney dysfunction might induce secondary hyperparathyroidism, resulting in elevated PTH levels [36]. Furthermore, the deregulation of RAAS is typical in HF patients due to neuro-hormonal alterations observed in this pathological condition and the use of dedicated pharmacological treatments. RAAS alteration could impact the regulation of calcium excretion and reabsorption from the kidney, thus promoting a further mechanism for PTH increase [34]. Our study outlined that the increase in PTH plasma levels with the reduction in eGFR values and eGFR still remained an independent predictor of mortality in patients with HF (Figure 3B and Table 2).

Although PTH seemed to be influenced by sex when dealing with mean platelet volume (MPV)—a potential marker of platelet activation—in patients with symptomatic HF [37], our study outlined no impact of sex in the concentration of PTH in congested patients.

Therefore, although PTH is related to congestion in HF, thus providing further advice to physicians for the comprehension of the fluid overload status of patients with HF as a congestion biomarker, its prognostic impact is questionable and related to kidney function. Indeed, LVEF, eGFR, and multiparametric score for congestion identification provide interesting prognostic insight in the evaluation of all-cause mortality in HF patients. Our group has already demonstrated the prognostic implication of creatinine clearance in patients with HF [38] as well as the impact of congestion in the overall mortality rate in these patients [25].

Although reduced LVEF is known to be directly related to adverse events and prognosis in patients with HF [39], the identification of LVEF as an independent determinant of prognosis in agreement with congestion biomarkers further pointed out the need for a comprehensive evaluation of the fluid overload of patients with HF. As PTH was related to congestion and to the decrease in kidney function, PTH could be included in common evaluation of patients with HF in order to firstly promote a more intelligible identification of the fluid overload of patients and the impact of this on outcomes.

## 5. Limitations

This paper has some limitations. Firstly, we did not evaluate the presence of secondary hyperparathyroidism. As the main aim of the paper was to evaluate the impact of the serum level of PTH in patients with HF, we did not concentrate on the causes for secondary hyperparathyroidism, which could be the focus of further studies in the context of HF. The lack of end-stage kidney failure/dialysis patients and the demonstration of the independent value of our results from kidney function reduce the probability for secondary causes of hyperparathyroidism. Secondly, the retrospective nature of this study is a further limitation: dedicated cohort longitudinal studies might be managed in order to reinforce the results. Finally, this was a single-centre study: the involvement of several HF centres located at different sites might promote confirmation of the results.

## 6. Conclusions

PTH is a reliable biomarker for congestion status in patients with HF as it is related to peripheral oedema, orthopnoea, and HYDRA score, independently from renal function. PTH was not an independent predictor for all-cause mortality in HF but might contribute to the correct evaluation of the congestion status of patients with both AHF and CHF.

## Figures and Tables

**Table 1:** Patient characteristics.

	*n* = 228
Clinical characteristics	
Age, yrs	75 ± 0.7
Male, %	52
BMI, kg/m^2^	28 ± 0.3
NYHA class II/III/IV, *n*	98/69/61
Medical history, %	
Coronary artery disease	28
Diabetes	21
Atrial fibrillation	43
PM/ICD	16
AHF	44
LVEF, %	42 ± 0.8
Clinical congestion	
Peripheral oedema, %	26
Orthoponea, %	27
Laboratory values	
PTH, pg/dL	191 ± 10
Haemoglobin, g/dL	13 ± 0.1
Uric acid, mg/dL	6.4 ± 0.1
BUN, mg/dL	31 ± 2.4
Creatinine, mg/dL	1.4 ± 0.04
eGFR, mL/min/1.73 m^2^	53 ± 1.8
Sodium, mmol/L	139 ± 0.2
Potassium, mmol/L	4.0 ± 0.04
Chloride, mmol/L	103 ± 0.3
Calcium, mmol/L	8.7 ± 0.05
Inorganic phosphorus (mg/dL)	3.6 ± 0.07
Albumin, g/dL	3.3 ± 0.04
Corrected calcium (mg/dL)	9.2 ± 0.05
Biomarkers of congestion	
BNP, pg/mL	978 ± 78
ePVS, dL/g	5.0 ± 0.1
Hydration index, %	75 ± 0.3
BUN/Cr ratio	54 ± 1.1
HYDRA score	1.7 ± 0.08
Therapies, %	
Furosemide	69
Beta-blockers	50
ACE inhibitors/ARBs	59
MRAs	50
Digitalis	22
Ivabradine	5

Data are expressed as mean ± standard error of mean or number and percentages. Abbreviations: ACE: angiotensin-converting enzyme; AHF: acute heart failure; ARB: angiotensin receptor blocker; BMI: body mass index; BNP: brain natriuretic peptide; BUN: blood urea nitrogen; CHF: chronic heart failure; eGFR: estimate glomerular filtration rate; ePVS: estimated plasma volume status; ICD: implanted cardioverter/defibrillator; LVEF: left ventricular ejection fraction; MRAs: mineralocorticoid receptor antagonists; NYHA: New York Heart Association; PM: pacemaker; PTH: parathyroid hormone.

**Table 2:** Univariate and multivariate Cox proportional hazards survival analyses.

	Univariate Cox Regression Analysis		Adjusted Cox Regression Analysis		
	HR (95% CI)	*p*	HR (95% CI)	*p*	Wald
AHF vs. CHF	2.85 (1.58–5.12)	=0.005	0.67 (0.31–1.47)	=0.3	
Age, year	1.05 (1.02–1.09)	=0.001	0.94 (0.96–1.04.994)	=0.9	
NYHA class	1.75 (1.25–5.46)	=0.001	0.94 (0.59–1.52)	= 0.8	
LVEF, %	0.97 (0.94–0.99)	=0.005	0.97 (0.94–0.99)	=0.009	6.9
PTH, pg/mL	1.003 (1.002–1.005)	<0.0001	1.001 (0.999–1.003)	=0.15	
HYDRA score	2.13 (1.67–2.70)	<0.0001	1.96 (1.44–2.67)	<0.0001	18
Corrected calcium	1.15 (0.57–1.50)	=0.47			
eGFR, mL/min	0.97 (0.95–0.98)	<0.0001	0.98 (0.96–0.99)	=0.04	4.3

Abbreviations: AHF: acute heart failure; CHF: chronic heart failure; CI: confidential interval; eGFR: estimate glomerular filtration rate; HR: hazard ratio; LVEF: left ventricular ejection fraction; NYHA: New York Heart Association; PTH: parathyroid hormone.

## Data Availability

Data supporting the reported results can be obtained by directly contacting the corresponding author.

## References

[1] Gruson D., Buglioni A., Burnett J.C. (2014). PTH: Potential role in management of heart failure. Clin Chim Acta..

[2] Tomaschitz A., Ritz E., Pieske B., Rus-Machan J., Kienreich K., Verheyen N., Gaksch M., Grübler M., Fahrleitner-Pammer A., Mrak P. (2014). Aldosterone and parathyroid hormone interactions as mediators of metabolic and cardiovascular disease. Metabolism.

[3] Wolzt M., Schmetterer L., Dorner G., Zelger G., Entlicher J., Kapiotis S., Eichler H.G. (1997). Hemodynamic effects of parathyroid hormone-related peptide-(1-34) in humans. J. Clin. Endocrinol. Metab..

[4] Bosworth C., Sachs M.C., Duprez D., Hoofnagle A.N., Ix J.H., Jacobs D.R., Peralta C.A., Siscovick D.S., Kestenbaum B., de Boer I.H. (2013). Parathyroid hormone and arterial dysfunction in the multi-ethnic study of atherosclerosis. Clin. Endocrinol..

[5] Sugimoto T., Tanigawa T., Onishi K., Fujimoto N., Matsuda A., Nakamori S., Matsuoka K., Nakamura T., Koji T., Ito M. (2009). Serum intact parathyroid hormone levels predict hospitalisation for heart failure. Heart.

[6] Hagström E., Ingelsson E., Sundström J., Hellman P., Larsson T.E., Berglund L., Melhus H., Held C., Michaëlsson K., Lind L. (2010). Plasma parathyroid hormone and risk of congestive heart failure in the community. Eur. J. Heart Fail..

[7] Wannamethee S.G., Welsh P., Papacosta O., Lennon L., Whincup P.H., Sattar N. (2014). Elevated parathyroid hormone, but not vitamin D deficiency, is associated with increased risk of heart failure in older men with and without cardiovascular disease. Circ. Heart Fail..

[8] Meng F., Wang W., Ma J., Lin B. (2016). Parathyroid hormone and risk of heart failure in the general population: A meta-analysis of prospective studies. Medicine.

[9] Schierbeck L.L., Jensen T.S., Bang U., Jensen G., Køber L., Jensen J.E. (2011). Parathyroid hormone and vitamin D--markers for cardiovascular and all cause mortality in heart failure. Eur. J. Heart Fail..

[10] Bansal N., Zelnick L., Robinson-Cohen C., Hoofnagle A.N., Ix J.H., Lima J.A., Shoben A.B., Peralta C.A., Siscovick D.S., Kestenbaum B. (2014). Serum parathyroid hormone and 25-hydroxyvitamin D concentrations and risk of incident heart failure: The Multi-Ethnic Study of Atherosclerosis. J. Am. Heart Assoc..

[11] Hassan M., Qureshi W., Sroujieh L.S., Albashaireh D., BouMalham S., Liroff M., Amjad W., Khalid F., Hadid H., Alirhayim Z. (2014). Interplay of parathyroid hormone and aldosterone antagonist in prevention of heart failure hospitalizations in chronic kidney disease. J. Renin Angiotensin Aldosterone Syst..

[12] Sugimoto T., Dohi K., Onishi K., Yamada T., Horiguchi M., Takamura T., Kawamura A., Seko T., Nakamura M., Kasai A. (2014). Prognostic value of serum parathyroid hormone level in acute decompensated heart failure. Circ. J..

[13] Anderson J.L., Vanwoerkom R.C., Horne B.D., Bair T.L., May H.T., Lappé D.L., Muhlestein J.B. (2011). Parathyroid hormone, vitamin D, renal dysfunction, and cardiovascular disease: Dependent or independent risk factors?. Am. Heart J..

[14] Meems L.M., Brouwers F.P., Joosten M.M., Lambers Heerspink H.J., de Zeeuw D., Bakker S.J., Gansevoort R.T., van Gilst W.H., van der Harst P., de Boer R.A. (2016). Plasma calcidiol, calcitriol, and parathyroid hormone and risk of new onset heart failure in a population-based cohort study. ESC Heart Fail..

[15] Gruson D., Ferracin B., Ahn S.A., Zierold C., Blocki F., Hawkins D.M., Bonelli F., Rousseau M.F. (2015). 1,25-Dihydroxyvitamin D to PTH(1-84) Ratios Strongly Predict Cardiovascular Death in Heart Failure. PLoS ONE.

[16] Zhang S., Hu Y., Zhou L., Chen X., Wang Y., Wu J., He H., Gao Y. (2015). Correlations between serum intact parathyroid hormone (PTH) and N-terminal-probrain natriuretic peptide levels in elderly patients with chronic heart failure (CHF). Arch. Gerontol. Geriatr..

[17] Kolaszko A., Nowalany-Kozielska E., Ceranowicz P., Morawiec B., Kubiak G. (2018). The Role of Parathyroid Hormone and Vitamin D Serum Concentrations in Patients with Cardiovascular Diseases. Dis. Markers.

[18] Wu G., Wang X., Wang X., Jiang H., Wang L., Wang T., Liu J., An D., Cao L., Xia Y. (2016). Serum Parathyroid Hormone Levels Predict Discharge and Readmission for Heart Failure. Genet. Test Mol. Biomarkers.

[19] Gruson D., Lepoutre T., Ahn S.A., Ketelslegers J.M., Rousseau M.F. (2012). Increased circulating concentrations of bioactive PTH 1-84 in patients with heart failure. J. Endocrinol. Investig..

[20] Loncar G., Bozic B., Dimkovic S., Prodanovic N., Radojicic Z., Cvorovic V., Putnikovic B., Popovic V. (2011). Association of increased parathyroid hormone with neuroendocrine activation and endothelial dysfunction in elderly men with heart failure. J. Endocrinol. Investig..

[21] Gruson D., Ahn S.A., Rousseau M.F. (2015). Multiple biomarker strategy based on parathyroid hormone and natriuretic peptides testing for improved prognosis of chronic heart failure. Peptides.

[22] Altay H., Zorlu A., Binici S., Bilgi M., Yilmaz M.B., Colkesen Y., Erol T., Muderrisoglu H. (2012). Relation of serum parathyroid hormone level to severity of heart failure. Am. J. Cardiol..

[23] Wu G.Y., Shen Q., Wu T., Shi Y.C., Wang T.X., Zong G.J., Yang X.J. (2020). Serum parathyroid hormone levels in patients with chronic right heart failure. Biomed. Rep..

[24] Sugimoto T., Dohi K., Onishi K., Watanabe K., Sato Y., Sugiura E., Nakamori S., Nakajima H., Nakamura M., Ito M. (2013). Interrelatiosseonship between haemodynamic state and serum intact parathyroid hormone levels in patients with chronic heart failure. Heart.

[25] Massari F., Scicchitano P., Iacoviello M., Passantino A., Guida P., Sanasi M., Piscopo A., Romito R., Valle R., Caldarola P. (2020). Multiparametric approach to congestion for predicting long-term survival in heart failure. J. Cardiol..

[26] McDonagh T.A., Metra M., Adamo M., Gardner R.S., Baumbach A., Böhm M., Burri H., Butler J., Čelutkienė J., Chioncel O. (2022). 2021 ESC Guidelines for the diagnosis and treatment of acute and chronic heart failure: Developed by the Task Force for the diagnosis and treatment of acute and chronic heart failure of the European Society of Cardiology (ESC). With the special contribution of the Heart Failure Association (HFA) of the ESC. Eur. J. Heart Fail..

[27] Dickerson R.N., Alexander K.H., Minard G., Croce M.A., Brown R.O. (2004). Accuracy of methods to estimate ionized and “corrected” serum calcium concentrations in critically ill multiple trauma patients receiving specialized nutrition support. JPEN J. Parenter. Enter. Nutr..

[28] Cockcroft D.W., Gault M.H. (1976). Prediction of creatinine clearance from serum creatinine. Nephron.

[29] Matsue Y., van der Meer P., Damman K., Metra M., O’Connor C.M., Ponikowski P., Teerlink J.R., Cotter G., Davison B., Cleland J.G. (2017). Blood urea nitrogen-to-creatinine ratio in the general opulationandin patients with acute heart failure. Heart.

[30] Parrinello G., Torres D., Testani J.M., Almasio P.L., Bellanca M., Pizzo G., Cuttitta F., Pinto A., Butler J., Paterna S. (2015). Blood urea nitrogen to creatinine ratio is associated with congestion and mortality in heart failure patients with renal dysfunction. Intern. Emerg. Med..

[31] Duarte K., Monnez J.M., Albuisson E., Pitt B., Zannad F., Rossignol P. (2015). Prognostic Value of Estimated Plasma Volume in Heart Failure. JACC Heart Fail..

[32] Massari F., Iacoviello M., Scicchitano P., Mastropasqua F., Guida P., Riccioni G., Speziale G., Caldarola P., Ciccone M.M., Di Somma S. (2016). Accuracy of bioimpedance vector analysis and brain natriuretic peptide in detection of peripheral edema in acute and chronic heart failure. Heart Lung.

[33] Massari F., Mastropasqua F., Guida P., De Tommasi E., Rizzon B., Pontraldolfo G., Pitzalis M.V., Rizzon P. (2001). Whole-body bioelectrical impedance analysis in patients with chronic heart failure: Reproducibility of the method and effects of body side. Ital. Heart J..

[34] Bosselmann H., Tonder N., Sölétormos G., Gaborit F., Rossing K., Iversen K., Goetze J.P., Gustafsson F., Schou M. (2017). Influence of renal impairment on aldosterone status, calcium metabolism, and vasopressin activity in outpatients with systolic heart failure. ESC Heart Fail..

[35] Scagliola R., Brunelli C. (2022). Venous Congestion and Systemic Hypoperfusion in Cardiorenal Syndrome: Two Sides of the Same Coin. Rev. Cardiovasc. Med..

[36] Evenepoel P., Bover J., Ureña Torres P. (2016). Parathyroid hormone metabolism and signaling in health and chronic kidney disease. Kidney Int..

[37] Dahlen B., Müller F., Tröbs S.O., Heidorn M.W., Schulz A., Arnold N., Hermanns M.I., Schwuchow-Thonke S., Prochaska J.H., Gori T. (2021). Sex-Specific Relationship Between Parathyroid Hormone and Platelet Indices in Phenotypes of Heart Failure-Results From the MyoVasc Study. Front. Cardiovasc. Med..

[38] Scicchitano P., Iacoviello M., Passantino A., Guida P., De Palo M., Piscopo A., Gesualdo M., Caldarola P., Massari F. (2021). The Prognostic Impact of Estimated Creatinine Clearance by Bioelectrical Impedance Analysis in Heart Failure: Comparison of Different eGFR Formulas. Biomedicines.

[39] Okuhara Y., Asakura M., Orihara Y., Morisawa D., Matsumoto Y., Naito Y., Tsujino T., Ishihara M., Masuyama T. (2019). Reduction in Left Ventricular Ejection Fraction is Associated with Subsequent Cardiac Events in Outpatients with Chronic Heart Failure. Sci. Rep..

